# Development of a novel Newcastle disease virus (NDV) neutralization test based on recombinant NDV expressing enhanced green fluorescent protein

**DOI:** 10.1186/s12985-017-0900-8

**Published:** 2017-11-23

**Authors:** Ana Chumbe, Ray Izquierdo-Lara, Katherine Calderón, Manolo Fernández-Díaz, Vikram N. Vakharia

**Affiliations:** 1 FARVET S.A.C, Carretera Panamericana Sur N° 766 Km 198.5, Chincha Alta, 11702 Ica, Peru; 2 Universidad Nacional Mayor de San Marcos, School of Veterinary Medicine, San Borja, Lima, Peru; 3 Institute of Marine & Environmental Technology, University of Maryland Baltimore County, 701 East Pratt St, Baltimore, MD 21202 USA

**Keywords:** Newcastle disease virus, Virus neutralization, Serum, eGFP

## Abstract

**Background:**

Newcastle disease is one of the most important infectious diseases of poultry, caused by Newcastle disease virus (NDV). This virus is distributed worldwide and it can cause severe economic losses in the poultry industry due to recurring outbreaks in vaccinated and unvaccinated flocks. Protection against NDV in chickens has been associated with development of humoral response. Although hemagglutination inhibition (HI) assay and ELISA do not corroborate the presence of neutralizing antibodies (nAbs); they are used to measure protection and immune response against NDV.

**Methods:**

In this study, we established a system to recover a recombinant NDV (rLS1) from a cloned cDNA, which is able to accept exogenous genes in desired positions. An enhanced green fluorescent protein (eGFP) gene was engineered in the first position of the NDV genome and we generated a recombinant NDV carrying eGFP. This NDV- eGFP reporter virus was used to develop an eGFP-based neutralization test (eGFP-NT), in which nAbs titers were expressed as the reciprocal of the highest dilution that expressed the eGFP.

**Results:**

The eGFP-NT gave conclusive results in 24 h without using any additional staining procedure. A total of 57 serum samples were assayed by conventional neutralization (NT) and eGFP-NT. Additionally, HI and a commercial ELISA kit were evaluated with the same set of samples. Although HI (*R* ^2^ = 0.816) and ELISA (*R* ^2^ = 0.791) showed substantial correlation with conventional NT, eGFP-NT showed higher correlation (*R* ^2^ = 0.994), indicating that eGFP-NT is more accurate method to quantify nAbs.

**Conclusions:**

Overall, the neutralization test developed here is a simple, rapid and reliable method for quantitation of NDV specific nAbs. It is suitable for vaccine studies and diagnostics.

**Electronic supplementary material:**

The online version of this article (10.1186/s12985-017-0900-8) contains supplementary material, which is available to authorized users.

## Background

Newcastle disease virus (NDV) is the causal agent of a highly contagious and fatal disease that affects poultry and other avian species worldwide [1]. Virulent strains of NDV are capable of causing high mortality (up to 100%) in non-vaccinated chickens [1]. NDV is a member of the genus *Avulavirus* of the family Paramyxoviridae in the order of Mononegavirals [2]. This virus has a nonsegmented single-stranded negative-sense RNA genome, which contains a 3′- leader and a 5′- trailer sequences, essential for virus transcription and replication, and follows the rule of six [3]. NDV possess six structural genes: Nucleoprotein (N), phosphoprotein (P), matrix (M), fusion (F), hemagglutinin-neuraminidase (HN) and large polymerase (L) [4]. From these proteins, N, P and L proteins form the Ribonucleoprotein (RNP) complex, which is responsible for viral transcription and replication [5]. HN and F are anchored in the viral envelope as surface glycoproteins: HN is responsible for the attachment of the virus to the host cell receptor, and F mediates fusion of the viral envelope with the host cell membrane [6]. The F protein is proteolytically cleaved to F1 and F2 for fusion activity and the presence of a polybasic motif in the cleavage site is a major determinant of virulence [6, 7]. Both HN and F proteins are capable of eliciting neutralizing antibodies (nAbs) [8–12].

Humoral immunity plays an essential role in the protection against NDV infection. Chickens with high antibody titers are usually protected. For example, young chicks with high maternal antibody titers are protected against a challenge with a virulent strain during the first few days [12]. Protection against the virus has been described for chickens passively immunized with egg yolk or antiserum from hyperimmunized birds against the whole virion. Monoclonal antibodies against HN and F proteins are able to neutralize the virus, both in vitro and in vivo [13–15]. Although, antibodies against F and HN have a synergistic potential [14]. Recently, higher and specific levels of antibodies were not only related with protection against mortality, but also with reduction of viral replication and secretion [16]. Hence, measuring the neutralizing antibodies (nAbs) against NDV is highly essential to evaluate the efficacy of a vaccine.

Usually, hemagglutination inhibition (HI) assay and Enzyme-Linked ImmunoSorbent Assay (ELISA) are used to measure NDV-specific antibodies but not necessarily nAbs against NDV. Conventional neutralization test (NT) is laborious, time-consuming and may have operator bias. Therefore, a rapid, high-throughput and reliable NT assay is necessary for evaluation of NDV nAbs. In recent years, few researchers have shown that genetically engineered viruses expressing the green fluorescent protein (GFP) or the enhanced GFP (eGFP) can be used for rapid determination of virus neutralizing antibody titers or antiviral activities [17–21]. The eGFP expressed by these viruses allows direct visualization of the infection under a fluorescent microscope or its automatization by using a fluorescence reader plate. These characteristics make it a suitable method to overcome the drawbacks of a conventional NT.

In this report, we describe the generation of a genetically engineered NDV expressing the eGFP from cDNA, and development of an eGFP-based NT (eGFP-NT) for rapid detection of NDV nAbs. Our results show that this method is fairly accurate as a conventional NT method but a better alternative in terms of being cost-effective and efficient.

## Methods

### Cell lines

Two cell lines were used in this study, DF-1 (derived from chicken fibroblasts) and Vero (monkey kidney cells), which were purchased from ATCC (Manassas, VA, USA). Both cell lines were maintained in Dulbecco’s modified Eagle medium (DMEM) F12 (HyClone) supplemented with 5% heat-inactivated fetal bovine serum (FBS), 2.5% chicken serum (ChkS) (Sigma–Aldrich), 100 U/mL of penicillin and 100 μg/mL of streptomycin at 37 °C in an atmosphere of 5% CO_2_.

### Construction of a full-length clone of NDV

**Fig. 1:**
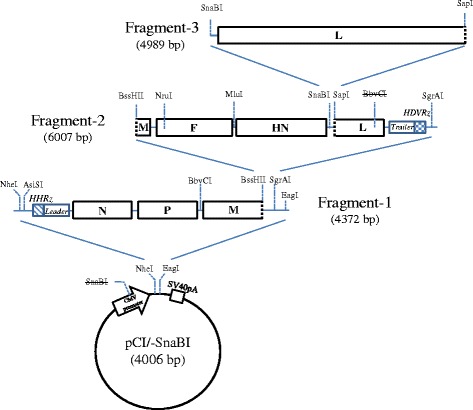
Construction of the full-length clone rLS1. Three fragments covering the entire NDV genome were assembled by successive ligation into the modified pCI/−SnaBI plasmid. Fragments-1 and -2 were chemically synthetized, whereas, fragment-3 was excised from pNDV-PE18 plasmid. Fragment-1 contains the hammerhead ribozyme (HHRz) at the 5′-end, the leader sequence, and N, P and partial M genes. Fragment-2 contains the partial M, F, HN and partial L genes, the trailer sequence and the hepatitis delta virus ribozyme (HDVRz). Fragment-3 contains the remaining portion of the L gene. Cross out restriction sites SnaBI and BbvCI indicate that these sites were destroyed with regard to their original sequences

Based on the published nucleotide sequence of lentogenic NDV strain in the GenBank (Accession No. Y18898) [22], a full-length clone of NDV (pFLC-LS1) was assembled in pCI-modified vector (pCI/−SnaBI) from three overlapping fragments. Figure 1 shows sequential subcloning of three fragments into the vector, each having unique restriction sites, namely NheI-EagI, BssHII-SgrAI, and SnaBI-SapI, respectively. Fragments-1 and -2 were chemically synthesized (GenScript, New Jersey, USA) that contain NDV sequences, and fragment-3 was taken from previously recovered full-length pNDV-PE18 [23], which was derived from LaSota strain. Fragment-1 (4387 nt) includes sequences (from 5′ to 3′) of a self-cleaving hammerhead ribozyme (HHRz), the N, P and partial M genes, between the NheI and EagI restriction sites. The HHRz sequence 5′-CTG ATG AGT CCG TGA GGA CGA AAC TAT AGG AAA GGA ATT CCT ATA GTC-3′ was included immediately upstream of the Leader sequence of the virus. Fragment-2 (5865 nt) consists of partial M, F, HN and partial L protein genes and the hepatitis delta virus anti-genome ribozyme (HDVRz). The HDVRz sequence 5′-GGG TCG GCA TGG CAT CTC CAC CTC CTC GCG GTC CGA CCT GGG CAT CCG AAG GAG GAC AGA CGT CCA CTC GGA TGG CTA AGG GAG AGC CA-3′ was inserted immediately downstream of the Trailer sequence of the virus [24]. Fragment-3 (4989 nt) contains the remaining L gene sequence (4769 nt upstream) between SnaBI and SapI restriction sites. All nucleotide changes due to creation or deletion of restriction sites in the clone are shown in Table 1. Finally, the resulting plasmid pFLC-LS1 (19,319 bp) was obtained which contains the plus sense genome sequence of NDV (15,186 nt). DNA of this plasmid was completely sequenced to verify its integrity by Sanger sequencing (Macrogen Inc., Korea).

**Table 1:** Nucleotide differences between the reference NDV and the assembled full-length clone, pFLC-LS1

Nucleotide Position^a^	Nucleotide change		Gene	Observation^c^
	Reference	Full-length clone		
4291	A	G	M	Creation of BssHII
4552	CA	GC	F	Creation of NruI
6294	CG	GC	F-HN^b^	Creation of MluI
8352	CAGCTC	TACGTA	HN-L^b^	Creation of SnaBI
8817	A	G	L	per se mutation
9440	T	C	L	per se mutation
11,657	G	A	L	per se mutation
12,567	C	T	L	per se mutation
14,608	T	A	L	Destruction of BbvCI

^a^ Numbering of the nucleotide position is relative to the reference NDV (GenBank sequence no. Y18898)

^b^Intergenic regions between F and HN or between HN and L genes, respectively

^c^Differences in the cDNA sequence were caused by introduction/destruction of artificial restriction sites (in fragments-1 and -2) or per se the genome sequence (in fragment-3)

### Construction of supporting plasmids

Supporting plasmids were generated from the full-length clone pFLC-LS1 vector using primers Nfor, Nrev, Pfor, Prev, Lfor and Lrev (Table 2). Amplified open reading frames (ORFs) of the N, P and L protein genes were cloned into the pCI vector. The resulting plasmids pCI-N, pCI-P and pCI-L were used for the recovery of recombinant viruses. The ORFs of N and P proteins were amplified by PCR using gene-specific primers that contain EcoRI and NotI restriction sites, whereas the L ORF was amplified using Lfor and Lrev primers that contain SpeI and NotI restriction sites, respectively. The L ORF was cloned between NheI and NotI, which resulted in the destruction of NheI/SpeI restriction site. All forward primers contained the Kozak sequence GCCACC immediately upstream of the start codon (ATG).

**Table 2:** Oligonucleotides used for cloning of the supporting plasmids, construction of pFLC-LS1-1eGFP, and confirmation of virus recovery

Primers	Sequences (5′ → 3′)^a,b^	Restriction site	Nucleotide Position^c^
Supporting plasmid primers			
Nfor	GGAATTC **GCCACC**ATGTCTTCCGTATTTGATG	EcoRI	116–140
Nrev	GCGGCCGCTCAATACCCCCAGTCGGTGT	NotI	1572–1591
Pfor	TGAATTC **GCCACC**ATGGCCACCTTTACAGATGC	EcoRI	1886–1906
Prev	GCGGCCGCTTAGCCATTTAGAGCAAG	NotI	3057–3074
Lfor	TACTAGT **GCCACC**ATGGCGAGCTCCGGTCCTGAA	SpeI	8380–8401
Lrev	GCGGCCGCTTAAGAGTCACAGTTACTGT	NotI	14,976–14,995
pFLC-LS1-1eGFP construction			
NDV116_F	GCCAACATGTCTTCCGTATTTGATG	PciI	116–140
NDV3211_R	CGATCATTCAGTGGGGCTGAGG	BbvCI	3190–3211
Confirmation primers			
M + 4100 ^d^	AGTGATGTGCTCGGACCTTC	N.A.^e^	4100–4119
NDV4394_R	GAGACGCAGCTTATTTCTTAAAAGG	N.A.	4370–4394
NDV14035_F	TCCAGGGTCCAATCAAAGCT	N.A.	14,035–14,054
NDV15003_R	GATTTTCGTTAAGAGTCACAGTTACTG	N.A.	14,977–15,003

^a^Restriction sites sequences are underlined

^b^Kozak sequence is shown in bold

^c^The positions where primers bind are according to the published NDV sequence (GenBank Accession no. Y18898)

^d^This primer was taken from Wise et al. [26]

^e^Not applicable

### Construction of pFLC-LS1-1eGFP plasmid

**Fig. 2:**
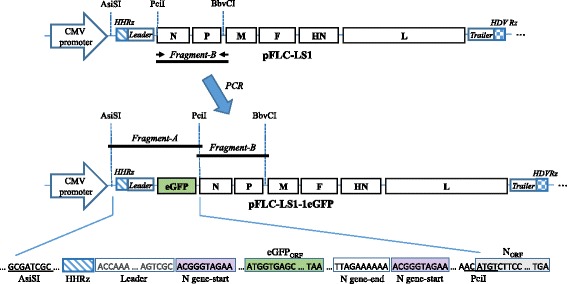
Cloning strategy to incorporate the eGFP gene into the full-length NDV clone rLS1. Three fragments ligation reaction was performed to insert the eGFP gene into the rLS1 genome between AsiSI and BbvCI restriction sites. Fragment-A containing the eGFP cassette was chemically synthetized, whereas, fragment-B was amplified by PCR using the pFLC-LS1 clone as a template. Both fragments were simultaneously cloned into the pFLC-LS1 to generate the pFLC-LS1-1eGFP. The final construct includes the eGFP ORF, flanked by gene-start and gene-end from the N gene, inserted at the 3′- proximal position of the NDV genome

To insert the eGFP gene in the NDV full-length clone, fragment-A was chemically synthesized (GenScript, New Jersey, USA). Fragment A flanked by AsiSI and PciI restriction sites contains the HHRz ribozyme sequence, Leader sequence, eGFP gene cassette flanked by the gene-start and gene-end of the N gene and right after the gene-end a second gene-start that will be part of the N cassette as shown in Fig. 2. To achieve high levels of expression, the eGFP cassette was inserted into the most 3′-proximal locus [25] between AsiSI and BbvCI sites of the clone by ligation with fragment-B (Fig. 2). Fragment-B contains the N and P genes flanked by PciI and BbvCI restriction sites. This fragment was obtained by PCR amplification with primers NDV116_F and NDV3211_R (see Table 2), using the pFLC-LS1 as a template. Both fragments were cloned simultaneously in pFLC-LS1 between AsiSI and BbvCI sites (Fig. 2). The resulting plasmid was designated as pFLC-LS1-1eGFP which contains the NDV genome and eGFP gene that is 16,182 nt long, accomplishing the rule of six. DNA of this plasmid was completely sequenced to verify its integrity by Sanger sequencing (Macrogen Inc., Korea).

### Transfection and virus recovery

High-quality plasmid DNA was obtained from the pFLC-LS1, pFLC-LS1-1eGFP and three supporting plasmids using the Plasmid Midi Kit (Qiagen). Vero cells were grown to 80% confluency in a 12-well plate and then co-transfected with 1 μg of pCI-N, 0.5 μg of pCI-P, 0.2 μg of pCI-L and 2 μg of pFLC-LS1 or pFLC-LS1-1eGFP, using the Lipofectamine™ LTX & plus Reagent (Invitrogen, USA) according to manufacturer’s instructions. Briefly, plasmids were diluted in a 400 μl of Opti-MEM medium (Invitrogen, USA). Next, Lipofectamine was added and incubated for 15 min at room temperature. Vero cells were washed with Dulbecco’s Phosphate Buffered Saline (DPBS) and then the plasmid-Lipofectamine mixture was added to the monolayer. After 4 h of incubation at 37 °C, transfected cells were washed and then maintained in DMEM containing 5% FBS at 37 °C and 5% CO_2_. Next day, allantoic fluid (AF) was added to a final concentration of 5% and cells were incubated for 4 more days. Cell supernatant was harvested, pelleted at high speed and inoculated into 8-days old SPF chicken embryonated eggs and incubated for 4 days. AFs were harvested, clarified, and aliquoted and stored at −80 °C. Recovery of the viruses was first confirmed by hemagglutination assay (HA). For parental virus (rLS1), the presence of NDV-specific protein was evaluated by immunofluorescence and confirmation of genetic markers was performed by RT-PCR and restriction enzyme digestions. To confirm the recovery of rLS1-1eGFP virus, infected DF-1 cells were observed under a fluorescence microscope.

### Immunofluorescence of rLS1

DF1 cells infected with the rLS1 virus at a multiplicity of infection (MOI) of 0.01 for 48 h. After discarding the supernatant, the cells were washed with DPBS 3 times (as all washing steps in this section) and fixed with 4% paraformaldehyde in DPBS for 15 min at room temperature. Fixed cells were washed, permeabilized with 1% SDS in DPBS for 15 min at room temperature and washed again. Permeabilized cells were blocked with 5% Bovine Serum Albumin (BSA) (Sigma-Aldrich) in DPBS for 30 min at room temperature. Cells were incubated for 2 h with a monoclonal antibody against the NDV ribonucleoprotein (RNP) (cat. n° ab138719, Abcam, USA) at a final concentration of 3.85 μg/mL in a solution of 5% BSA in DPBS. After washing, the cells were incubated for 1 h with a goat polyclonal antibody anti-mouse IgG labeled with Alexa Fluor 594 (cat n° ab150116, Abcam) at a final concentration of 1 μg/mL in a solution of 5% BSA in DPBS, then washed. Nuclei were stained with DAPI for 5 min. Cells were examined under the Observer.A1 fluorescent microscope (Carl Zeiss, Germany). The fluorescent signal images were taken at 400X magnification with the AxioCam MRc5 camera (Carl Zeiss, Germany).

### RT-PCR and confirmation of genetic markers

The identity of genetic markers in recombinant NDVs was confirmed after RT-PCR amplification from genomic RNA and sequencing of the DNA fragments. Two genetic markers evaluated were; creation of the BssHII site in the M gene and destruction of the BbvCI site in the L gene (see Table 1). Briefly, viral RNA was extracted from AFs stocks using the QIAamp Viral RNA mini kit (Qiagen) and cDNA was generated using the ProtoScript® II First Strand cDNA Synthesis Kit (New England Biolabs Inc). The PCR reactions were performed with primers M + 4100 [26] and NDV4394_R to amplify the region containing the BssHII site, and primers NDV14035_F and NDV15003_R for BbvCI site that was destroyed (see Table 2). Restriction analysis of the PCR products was carried out on a 2% agarose gel.

### Plaque assay

DF-1 cells were seeded into 12-well plates at density 1.5 × 105 cells in 1 mL of DMEM F12 per well, a day before the assay. Next day, serial dilutions of 1:10 were made by mixing 25 μL of virus with 225 μL of serum free DMEM F12. After washing DF-1 cells monolayer with DPBS, dilutions (0.2 mL/well) were added into wells and incubated for 1 h at 37 °C in an atmosphere of 5% CO_2_ for viral absorption. Later, the inoculum was removed and each well was covered with 1 mL of an overlay medium consisting of DMEM supplemented with 0.5% agarose, 5% AF and 30 mM MgCl_2_ [27]. The overlay medium was allowed to solidify by placing the plates on a level surface at room temperature for 15 min, the plates were then incubated for 5 days at 37 °C and 5% CO_2_. The plates were fixed and stained with a mixture of 0.2% crystal violet (Sigma-Aldrich) and 3.2% paraformaldehyde overnight at room temperature [28]. The plates were rinsed with tap water, dried, viewed and photographed for EliSpot (EliSpot Reader versión 7.0). The NDV titers were reported as plaque-forming units per milliliter (PFU/mL).

### Virus growth curves in DF-1 cells

Monolayer cultures of DF-1 cells were seeded at 50–60% confluence in 12-well plates and infected with rLS1 or rLS1- eGFP viruses at a (MOI) of 0.05. Cells were cultured with DMEM containing 1% FBS and 5% AF with 5% CO_2_ at 37 °C. Supernatants were collected 12, 24, 36, 48, 60 and 72 h post-infection (h.p.i.). Collected supernatants were quantified in DF-1 cells by plaque assay as described above.

### Serum samples

Serums samples obtained from 57 chickens were used for the experiments. Thirty seven serum samples were collected from field vaccinated chickens from different farms. Six commercially available chicken sera (Charles River Laboratories), corresponding to sera anti-Marek’s disease virus (MDV), anti-Avian adenovirus type-1, anti-Infectious Laryngotracheitis virus (ILTV), anti-Infectious bronchitis virus (IBV), anti-NDV, another anti-NDV serum (cat. n° ab34402, Abcam), and a serum from a SPF chicken was included as negative control. The remaining 13 serum samples were obtained from SPF chickens inoculated with one dose of LaSota vaccine at 1 day of age and their sera taken at the 4th week. All serum samples were heat inactivated (56 °C for 30 min) and stored at −20 °C [29]. Detailed information about the serum samples is listed in Additional file 1: Table S1.

### Neutralization tests

Conventional NT and eGFP-NT were carried out to quantify nAbs in chicken sera. For both assays, serum samples were titrated in duplicate, using 96-well flat bottom nonpyrogenic polystyrene culture plates (Corning, NY, USA). A day before the assay, 1 × 10^4^ DF-1 cells per well were seeded in 0.1 mL of DMEM F12 supplemented with 5% FBS. The serum samples were serially diluted by 2-fold (starting from 1:2) with DMEM F12 serum free medium, supplemented with FA and FBS at final concentrations of 5% and 1%, respectively. Serum dilutions were mixed with 100 PFU of virus (rLS1 or rLS1-1eGFP, respectively). Each dilution was evaluated in duplicate. For conventional NT, DF-1 cells were infected with mixtures of virus-serum. After 4 days of incubation at 37 °C in 5% CO_2_, cells were washed with DPBS. Then cells were fixed and stained with a mixture of 0.2% crystal violet and 3.2% paraformaldehyde for 15 min at room temperature. NDV nAb titers were determined as the reciprocal of the highest dilutions that both replicates presented a clear CPE.

For eGFP-NT, once DF-1 cells were infected with mixtures of virus-serum, cells were incubated for 48 h and observed under a fluorescence microscope and NDV nAb titers were determined as the reciprocal of the highest dilutions that did not express the eGFP in any of the duplicates. Wells with one or more fluorescent foci were considered positive [30].

### Hemagglutination inhibition (HI) test

HI was performed according to the OIE terrestrial manual [31]. Briefly, 25 μL of PBS per well were dispensed in clear 96-well V-bottom plates, then 25 μL of serum was placed on the first well, 2-fold dilutions of 25 μL of the serum suspension were made across the entire plate. Then, 25 μL of diluted virus containing 2^4^ hemagglutination units (HAU) of rLS1 virus was added to each well and incubated for 30 min at room temperature. Next, 25 μL of chicken red blood cells (RBCs) at 1% were added to each well and incubated for 40 min at room temperature. The titration was determined as the highest dilution of serum causing complete HI.

### Elisa

ELISAs against NDV (IDEXX laboratories, USA) were performed on all serum samples at room temperature, according to manufacturer’s instructions. Briefly, 100 μl of each serum sample (diluted 1:500 in DPBS), and 100 μl of the negative and positive control samples were dispensed into duplicate wells and incubated for 30 min. The plates were then washed thrice and incubated with 100 μl of conjugate per well for 30 min. Wells were washed thrice and then 100 μl per well of substrate solution was added, incubated in the dark for another 30 min, after which 100 μl stop solution was immediately added. The plates were read using an Epoch 2 microplate reader (Biotek, USA) at 450 nm. Data obtained were analyzed and sample to positive ratio (S/P) calculated.

### Statistical analysis

The t-student test was performed to compare the mean titer of each time of the growth kinetics curve. Linear regressions were performed for the analysis of correlation between NT and eGFP-NT, HI and ELISA titers. All statistical analysis were performed in GraphPad Prism 6.01 (GraphPad Software Inc., San Diego, CA).

## Results

### Construction and characterization of the recombinant rLS1 NDV

**Fig. 3:**
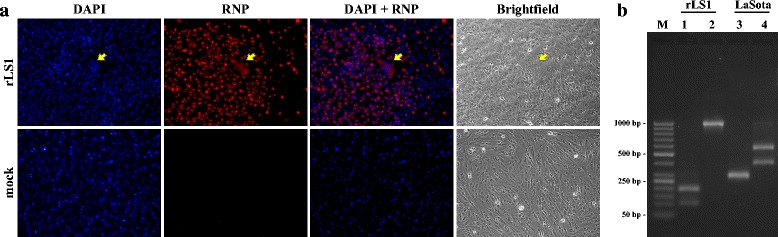
Identification of the rescued rLS1 virus. **a** DF-1 cells infected with rLS1 at an MOI of 0.01. After 48 h, the cells were fixed and the presence of NDV was detected using a NDV-specific monoclonal antibody against ribonucleoprotein. Infected cells showed cell fusion and formation of syncytia (yellow arrow) ×200. **b** Confirmation of the recovered rLS1 virus by RT-PCR and restriction digestion. Genomic RNA isolated from recovered rLS1 and LaSota viruses was amplified by RT-PCR with specific primers covering the created (lanes 1 and 3) or destroyed restriction sites (lanes 2 and 4), and the products analyzed on a 2% agarose gel. Lane M: 50 bp molecular size marker; lane 1: BssHII for rLS1 (192 bp and 103 bp); lane 2: BbvCI for rLS1 (969 bp); lane 3: BssHII for LaSota (295 bp); lane 4: BssHII for LaSota (573 bp and 396 bp)

The full-length NDV clone pFLC-LS1 was assembled together with three fragments through several restrictions sites, as depicted in Fig. 1. We used an RNA polymerase II promoter system to recover the virus, as previously reported for NDV [32, 33], which allowed the virus rescue in a T7 RNA polymerase independent fashion. Unique restriction sites created or destroyed by silent mutations into the full-length clone as genetic markers (see Table 1) were verified by sequencing the DNA. This full-length clone, along with the supporting plasmids, was used to transfect DF-1 cells. Three days after transfection, the cytopathic effect (CPE) was observed and recombinant rLS1 was recovered at a high titer after the first passage in chicken eggs (1 × 10^8^ PFU/mL). Immunofluorescence assay of infected cells was positive when reacted with NDV-specific monoclonal antibody against the RNP complex, confirming the presence of NDV (Fig. 3a). To verify the identity of the rLS1 virus and dismiss any possible contamination by other NDV strain i.e. LaSota, two of the genetic markers were assessed by RT-PCR and restriction enzyme digestion (Fig. 3b). Absence of BssHII site and deletion of BbvCI restriction site was verified by RT-PCR, followed by restriction enzyme digestion, using LaSota strain as a control (see Fig. 3b). In addition, sequencing of these amplified DNA fragments were performed in order to confirm the existence of genetic markers, proving that rLS1 virus was certainly derived from pFLC-LS1 plasmid.

### Construction and characterization of the rLS1-1eGFP reporter NDV

**Fig. 4:**
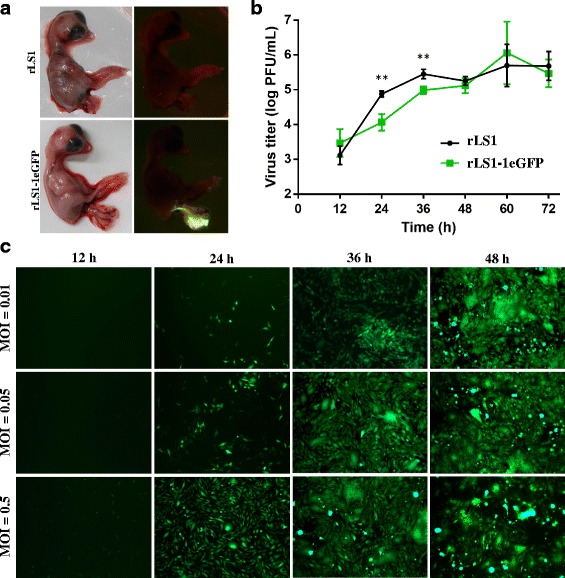
Characterization of rLS1-1eGFP virus. **a** SPF chicken embryos infected with rLS1 or rLS1-1eGFP. The embryos were inoculated at 9 days of age and incubated for 3 days. The eGFP expression was clearly observed at the umbilical cord of the embryos. **b** Comparison of the growth kinetics of rLS1 and rLS1-1eGFP. DF-1 cells were infected with an MOI of 0.05 and supernatant was collected in 12 h intervals post-infection and tittered by plaque assay. The data were taken from three independent experiments. Statistical significance (*p* < 0.01) was denoted with two asterisks. **c** Correlation between eGFP expression and different MOIs and times of infection. DF-1 cells were infected with rLS1-1eGFP at indicated MOIs and eGFP expression observed at 12, 24, 36 and 48 h.p.i

In order to obtain high expression levels of the eGFP, we inserted the eGFP cassette into the first position within the NDV genome. The pFLC-LS1-1eGFP construct was correctly assembled, as verified by sequencing of the entire plasmid, and used for transfection of DF-1 cells. Transfection supernatant containing the rLS1-1eGFP virus was passaged in SPF embryonated eggs. The collected AF contained a titer of 4.8 × 10^8^ PFU/mL, similar to the parental virus. Chicken embryos infected with the rLS1-1eGFP observed under a transilluminator showed high levels of eGFP expression in the umbilical cord (Fig. 4a). Replication in umbilical cord stem cells have also been reported for other viruses such as Influenza, hepatitis B and herpes simplex 1 viruses [34–36]. For NDV, since the chicken umbilical cord is connected with the chorioallantoic membrane, it is possible that umbilical cord stem cells allowed the replication of the virus at high levels and then release them into the allantoic fluid.

Comparison of growth kinetics of the recovered viruses was performed in DF-1 cells at an MOI of 0.05. Both viruses exhibited similar growth characteristics. However, rLS1-1eGFP showed significantly lower titers than rLS1 (*p* < 0.01) at 24 and 36 h.p.i. (Fig. 4b). The highest titers achieved in cell culture for both viruses were approximately 10^6^ PFU/ml (Fig. 4b), whereas the titers in AF collected from SPF embryonated eggs were at least 10^8^ PFU/ml (data not shown).

The correlation between eGFP expression level and the efficiency of viral replication was assessed by infecting DF-1 cells at different MOIs with rLS1-eGFP, and recording these cells every 12 h. The intensity and number of cells expressing the eGFP were increasing in a time- and dose-dependent manner (Fig. 4c). The eGFP expression can be clearly visualized as early as 24 h.p.i., although it can be detected with low expression at 12 h.p.i. These results indicates that eGFP expression can be used to monitor viral replication.

### Strong correlation between conventional NT and the eGFP-NT

Conventional- and eGFP-NT were first evaluated by using diluted reference chicken serum samples. Reference serum samples, positive to other pathogens (IBV, MDV, Avian adenovirus type 1 and ILTV) and from a SPF chicken, were used to evaluate non-specific neutralization. These sera tested negative, as expected, for both neutralization methods (See Additional file 1: Table S1). Fluorescence in the eGFP-NT was visible as early as 18 h of incubation, and it was clearly visible at 24 h.

**Fig. 5:**
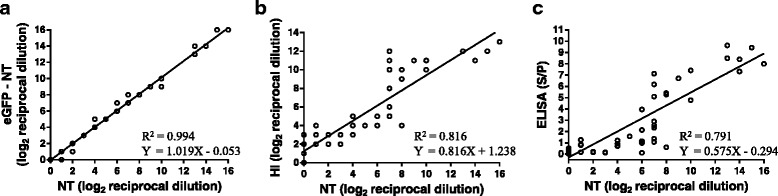
Comparison of conventional NT with eGFP-NT, HI and ELISA of 57 chicken serum samples. **a** Correlation of conventional NT and eGFP-NT. Neutralization titers (X-axis) are given as the log_2_ of the reciprocal serum dilution in which 100% CPE is inhibited. eGFP-NT titers (Y-axis) are given as the log_2_ of the maximal reciprocal serum dilution in which all wells show non-eGFP expression. **b** Correlation of conventional NT and HI. HI titers (Y-axis) are given as the log_2_ of the maximal reciprocal serum dilution in which hemagglutination is inhibited. **c** Correlation between NT titers (X-axis) and ELISA titers (Y-axis). ELISA is given as the S/P ratio

To assess the efficacy of rLS1-1eGFP virus to measure nAb titers, the conventional- and eGFP-NT were performed using a total of 57 serum samples. Most of the samples presented the same neutralization titer when measured with both techniques. Moreover, the correlation between the conventional NT and eGFP-NT was very high (*R* ^2^ = 0.994), as shown in Fig. 5a. Thus, we conclude that rLS1-1eGFP can be used as a reporter virus to determine nAbs titers with great reliability.

### HI and ELISA have strong correlations with conventional NT but present greater dispersion than eGFP-NT

Correlations between nAbs titers and the antibody titers measured by HI and ELISA were evaluated. In Fig. 5b, the relation between log2 HI and log2 NT is shown. A strong correlation (*R* ^2^ = 0.816) was observed between NT and HI titers. In addition, several samples that tested positive to the HI assay, with titers corresponding to dilutions of up to 1:8, were negative for neutralization. These results are consistent with the fact that the equation of the line (Y = 0.816X + 1.238) shows that when NT is 0, in average, HI titers would be positive and greater than a titer corresponding to a dilution of 1:2. It is worth mentioning that serum samples positive to HI and negative to the NT assay came from vaccinated chickens (see Additional file 1: Table S1), suggesting that either nAbs are not circulating at that time because plasma cells become memory B cells or specific antibodies against NDV exist, but these are not nAbs.

Antibody titers measured by ELISA (S/P) also have a strong correlation (*R* ^2^ = 0.791) with the log_2_ NT titers (Fig. 5c). Both HI and ELISA showed greater dispersion to the conventional NT than the eGFP-NT. These results indicate that HI and ELISA are not as reliable as eGFP-NT, showing that this method can close the gap between a rapid and accurate measurement of nAbs against NDV.

## Discussion

NDV is one of the major threats to the poultry industry worldwide and new genotypes and sub-genotypes are discovered every year [37]. In South America, the virus is currently circulating [38–41], and there is an urgent need to improve vaccines as well as biosafety and surveillance. The relevance of nAbs in protection and against viral shedding has been well established [8, 12, 16]. Thus, NDV vaccines should be capable of eliciting high nAb titers. A rapid and easy-to-perform method to measure nAbs will help to evaluate new vaccine candidates. In this context, we developed a new vector (rLS1), which can accept inserts in all intergenic regions. Later, the eGFP gene was incorporated before the N gene into the rLS1 virus genome, generating the rLS1-1eGFP. We chose that position to produce high levels of eGFP protein [25]. This virus was used to develop the eGFP-NT to measure nAbs.

In our hands, conventional NT took 4 days to distinguish infected wells from non-infected ones, which were difficult to read from stained neutralization plate wells. In contrast, results from eGFP-NT can be obtained within a day. We set 24 h.p.i. as a conservative time to observe infected cells to measure nAb titers because at this time eGFP expression is very obvious in positive wells. Nevertheless, we obtained the same nAb titers when measured at 18 h or later (data not shown). For conventional NT, the difficulty to clearly differentiate infected from non-infected wells in limiting dilutions can lead to an operator bias. In contrast, eGFP-NT is a reliable tool, in which fluorescence can be easily detected, minimizing operator bias.

Conventional NT exhibited stronger correlation with the eGFP-NT (*R* ^2^ = 0.994) than ELISA (*R* ^2^ = 0.791) or HI (*R* ^2^ = 0.816). Although our data suggest good correlations between nAbs and HI or ELISA, there is window of uncertainty at low titers in both cases. Based on our regressions, HI assays overestimate and ELISA underestimate NT titers. Furthermore, these equations can be heavily affected based on the origin of the immunogen responsible to elicit humoral response. Several groups have shown that antibody titers measured by ELISA or HI do not necessarily correspond with NDV nAb titers [8, 12, 13]. Our results also show that HI tends to produce positive results in some samples with negative nAb titers, and both HI and ELISA have a big dispersion with respect to NT. In contrast, eGFP-NT has evident advantage over HI or ELISA because it clearly shows the presence of nAbs induced after vaccination. It is highly probable that correlations will dramatically change when subunit or virus-vectored NDV vaccines are used for immunization because only some epitopes will be displayed, but they will not necessary induce the production of antibodies detected by HI or ELISA. For example, avian paramyxovirus type 3 vectors carrying either F or HN proteins or the combination of both vectors were capable of eliciting similar nAb titers against LaSota-NDV control, but failed to elicit ELISA titers [42]. In other study, virus-like particles (VLP) based vaccine of NDV F protein and influenza M1 protein has been tested in SPF chickens, which gives low levels of NDV ELISA titers but still shows that birds were fully protected [43]. Similar results have been observed in fowl pox vectored NDV vaccine [44] and in a baculovirus expressing the F protein [10].

Recently, an NDV-pseudotyped HIV was engineered to express both F and HN proteins from NDV along with a luciferase reporter protein, and this virus was used for neutralization assays [45]. In our case, we have designed the NDV vector in such a way that it can accept inserts in almost all intergenic regions and one can exchange F and HN genes from various donors. The pFLC-LS1-1eGFP plasmid, containing the reporter eGFP gene in the NDV backbone, has unique restriction sites flanking the F (BssHII and MluI) and HN (MluI and SnaBI) genes, which can be used to easily replace the genes of other genotype. Additionally, the use of a reporter NDV expressing the eGFP has some advantages when compared to pseudotyped lentivirus vector that was used for neutralization assays: 1) Production of rLS1-1eGFP virus is technically easy, as the virus grows to high titers in both cell culture and embryonated eggs, allowing us to prepare large viral stocks. In contrast, lentiviruses require a new transfection procedure each time to generate the reporter virus [20]. 2) As mentioned earlier, eGFP-NT is cheaper, and does not require additional reagents. In contrast, lentiviruses expressing the luciferase enzyme require luciferase reporter kits and a luminometer to be quantified. 3) rLS1-1eGFP can be used for high throughput assays by measuring eGFP positive cells with a fluorescence reader directly in plates without any staining [18, 20, 21]. 4) Finally, rLS1-1eGFP vector can be used to express foreign F and HN in its natural context instead of using a surrogate system that may not reflect the same entry mechanism and therefore infectious efficacy.

## Conclusions

We generated a recombinant NDV harboring the eGFP from cloned cDNA, and developed an eGFP-based neutralization test (eGFP-NT) for rapid detection and quantification of nAbs against NDV. This novel test is simple, inexpensive, and accurate, which makes it suitable to test new NDV vaccine candidates. The eGFP-NT can be implemented in laboratories with basic cell culture equipment and a florescent microscope. So far, it is the quickest method to evaluate NDV nAbs, with a higher correlation to the conventional neutralization test.

## Additional file

Additional file 1: (XLSX 17 kb)

## References

[1] Hines NL, Miller CL (2012). Avian paramyxovirus serotype-1: a review of disease distribution, clinical symptoms, and laboratory diagnostics. Vet Med Int.

[2] Amarasinghe GK, Bào Y, Basler CF, Bavari S, Beer M, Bejerman N (2017). Taxonomy of the order Mononegavirales: update 2017. Arch Virol.

[3] Peeters BP, Gruijthuijsen YK, de Leeuw OS, Gielkens AL (2000). Genome replication of Newcastle disease virus: involvement of the rule-of-six. Arch Virol.

[4] Zhao H, Peeters BPH (2003). Recombinant Newcastle disease virus as a viral vector: effect of genomic location of foreign gene on gene expression and virus replication. J Gen Virol..

[5] Lamb RA, Parks GD, Fields BN, Knipe DN, Howley PM (2007). Paramyxoviridae: the viruses and their replication. Fields Virology.

[6] Peeters BP, de Leeuw OS, Koch G, Gielkens AL (1999). Rescue of Newcastle disease virus from cloned cDNA: evidence that cleavability of the fusion protein is a major determinant for virulence. J Virol.

[7] Ogasawara T, Gotoh B, Suzuki H, Asaka J, Shimokata K, Rott R, Nagai Y (1992). Expression of factor X and its significance for the determination of paramyxovirus tropism in the chick embryo. EMBO J.

[8] Reynolds DL, Maraqa AD (2000). Protective immunity against Newcastle disease: the role of antibodies specific to Newcastle disease virus polypeptides. Avian Dis.

[9] Sun HL, Wang YF, Tong GZ, Zhang PJ, Miao DY, Zhi HD, Wang M, Wang M (2008). Protection of chickens from Newcastle disease and infectious laryngotracheitis with a recombinant fowlpox virus co-expressing the F, HN genes of Newcastle disease virus and gB gene of infectious laryngotracheitis virus. Avian Dis.

[10] Mori H, Tawara H, Nakazawa H, Sumida M, Matsubara F, Aoyama S, Iritani Y, Hayashi Y, Kamogawa K (1994). Expression of the Newcastle disease virus (NDV) fusion glycoprotein and vaccination against NDV challenge with a recombinant baculovirus. Avian Dis.

[11] Morgan RW, Gelb J, Schreurs CS, Lütticken D, Rosenberger JK, Sondermeijer PJ (1992). Protection of chickens from Newcastle and Marek’s diseases with a recombinant herpesvirus of turkeys vaccine expressing the Newcastle disease virus fusion protein. Avian Dis.

[12] Umino Y, Kohama T, Kohase M, Sugiura A, Klenk HD, Rott R (1987). Protective effect of antibodies to two viral envelope glycoproteins on lethal infection with Newcastle disease virus. Arch Virol.

[13] Meulemans G, Gonze M, Carlier MC, Petit P, Burny A, Long L (1986). Protective effects of HN and F glycoprotein-specific monoclonal antibodies on experimental Newcastle disease. Avian Pathol.

[14] Russell PH (1986). The synergistic neutralization of Newcastle disease virus by two monoclonal antibodies to its haemagglutinin-neuraminidase protein. Arch Virol.

[15] Umino Y, Kohama T, Sato TA, Sugiura A (1990). Protective effect of monoclonal antibodies to Newcastle disease virus in passive immunization. J Gen Virol..

[16] Miller PJ, Afonso CL, El Attrache J, Dorsey KM, Courtney SC, Guo Z, Kapczynski DR (2013). Effects of Newcastle disease virus vaccine antibodies on the shedding and transmission of challenge viruses. Dev Comp Immunol.

[17] Li Y, Shen L, Sun Y, Yuan J, Huang J, Li C, Li S, Luo Y, Qiu HJ (2013). Simplified serum neutralization test based on enhanced green fluorescent protein-tagged classical swine fever virus. J Clin Microbiol.

[18] Tang HB, ZL L, Wei XK, Zhong YZ, Zhong TZ, Pan Y, Luo Y, Liao SH, Minamoto N, Luo TRA (2015). Recombinant rabies virus expressing a phosphoprotein-eGFP fusion is rescued and applied to the rapid virus neutralization antibody assay. J Virol Methods.

[19] Matsubara K, Fujino M, Takeuchi K, Iwata S, Nakayama TA (2013). New method for the detection of neutralizing antibodies against mumps virus. PLoS One.

[20] Deng CL, Liu SQ, Zhou DG, Xu LL, Li XD, Zhang PT, et al. Development of neutralization assay using an eGFP Chikungunya virus. Viruses. 2016;8(7). 10.3390/v8070181 PMC4974516 27367716

[21] van Remmerden Y, Xu F, van Eldik M, Heldens JG, Huisman W, Widjojoatmodjo MN (2012). An improved respiratory syncytial virus neutralization assay based on the detection of green fluorescent protein expression and automated plaque counting. Virol J.

[22] Römer-Oberdörfer A, Mundt E, Mebatsion T, Buchholz UJ, Mettenleiter TC (1999). Generation of recombinant lentogenic Newcastle disease virus from cDNA. J Gen Virol..

[23] Chumbe A, Izquierdo-Lara R, Tataje L, Falconi-Agapito F, Fernández M, Vakharia VN. Rescue of lentogenic asymptomatic Peruvian Newcastle disease virus. In: Proceedings of the sixty-third Western poultry disease conference, Puerto Vallarta, Jalisco, Mexico, 2014;1:68–73.

[24] Ammayappan A, Lapatra SE, Vakharia VNA (2010). Vaccinia-virus-free reverse genetics system for infectious hematopoietic necrosis virus. J Virol Methods.

[25] Huang Z, Krishnamurthy S, Panda A, Samal SK (2001). High-level expression of a foreign gene from the most 3′-proximal locus of a recombinant Newcastle disease virus. J Gen Virol.

[26] Wise MG, Suarez DL, Seal BS, Pedersen JC, Senne DA, King DJ, Kapczynski DR, Spackman E (2004). Development of a real-time reverse-transcription PCR for detection of Newcastle disease virus RNA in clinical samples. J Clin Microbiol.

[27] Lomniczi B (1974). Plaque assay for avirulent (lentogenic) strains of Newcastle disease virus. Appl Microbiol.

[28] Kaur P, Lee RCH, Chu JJH (2016). Infectious viral quantification of Chikungunya virus-virus plaque assay. Methods Mol Biol.

[29] Wang Z, Mo C, Kemble G, Duke G (2004). Development of an efficient fluorescence-based microneutralization assay using recombinant human cytomegalovirus strains expressing green fluorescent protein. J Virol Methods.

[30] Yager ML, Moore SM, Rupprecht C, Nagarajan T (2015). The rapid fluorescent focus inhibition test. Current laboratory techniques in Rabies diagnosis, research and prevention, vol 2.

[31] Afonso CL, Miller PJ, Grund C, Koch G, Peeters B, Selleck PW, Srinivas GB. Newcastle disease (infection with Newcastle disease virus). In: World Organization for Animal Health, editor. Manual of diagnostic tests and vaccines for terrestrial animals, 7th ed. OIE; 2012. p. 555–574.

[32] Li BY, Li XR, Lan X, Yin XP, Li ZY, Yang B, Liu JX (2011). Rescue of Newcastle disease virus from cloned cDNA using an RNA polymerase II promoter. Arch Virol.

[33] Chellappa MM, Dey S, Gaikwad S, Pathak DC, Vakharia VN (2017). Rescue of a recombinant Newcastle disease virus strain R2B expressing green fluorescent protein. Virus Genes.

[34] Khatri M, Chattha KS (2014). Replication of influenza a virus in swine umbilical cord epithelial stem-like cells. Virulence.

[35] Huang Y, Yan Q, Fan R, Song S, Ren H, Li Y, Lan Y, Hepatitis B (2016). Virus replication in CD34+ hematopoietic stem cells from umbilical cord blood. Med Sci Monit.

[36] Zhuo C, Zheng D, He Z, Jin J, Ren Z, Jin F, Wang Y. HSV-1 enhances the energy metabolism of human umbilical cord mesenchymal stem cells to promote virus infection. Future Virol. 2017;12(7)

[37] Dimitrov KM, Ramey AM, Qiu X, Bahl J, Afonso CL. Temporal, geographic, and host distribution of avian paramyxovirus 1 (Newcastle disease virus). Infect Genet Evol 2016;39:22–34. 10.1016/j.meegid.2016.01.008 26792710

[38] Perozo F, Marcano R, Afonso CL (2012). Biological and phylogenetic characterization of a genotype VII Newcastle disease virus from Venezuela: efficacy of field vaccination. J Clin Microbiol.

[39] Diel DG, Susta L, Cardenas Garcia S, Killian ML, Brown CC, Miller PJ, Afonso CL (2012). Complete genome and clinicopathological characterization of a virulent Newcastle disease virus isolate from South America. J Clin Microbiol.

[40] Chumbe A, Izquierdo-Lara R, Tataje-Lavanda L, Figueroa A, Segovia K, Gonzalez R, Cribillero G, Montalvan A, Fernández-Díaz M, Icochea E. Characterization and sequencing of a genotype XII Newcastle disease virus isolated from a peacock (Pavo Cristatus) in Peru. Genome Announc. 2015;3(4). 10.1128/genomeA.00792-15 PMC4520891 26227592

[41] Chumbe A, Izquierdo-Lara R, Tataje L, Gonzalez R, Cribillero G, González AE, Fernández-Díaz M, Icochea E (2017). Pathotyping and phylogenetic characterization of Newcastle disease viruses isolated in Peru: defining two novel subgenotypes within genotype XII. Avian Dis.

[42] Kumar S, Nayak B, Collins PL, Samal SK (2011). Evaluation of the Newcastle disease virus F and HN proteins in protective immunity by using a recombinant avian paramyxovirus type 3 vector in chickens. J Virol.

[43] Park JK, Lee DH, Yuk SS, Tseren-Ochir EO, Kwon JH, Noh JY (2014). Virus-like particle vaccine confers protection against a lethal Newcastle disease virus challenge in chickens and allows a strategy of differentiating infected from vaccinated animals. Clin Vaccine Immunol.

[44] Taylor J, Christensen L, Gettig R, Goebel J, Bouquet JF, Mickle TR, Paoletti E (1996). Efficacy of a recombinant fowl pox-based Newcastle disease virus vaccine candidate against velogenic and respiratory challenge. Avian Dis.

[45] Wang B, Wang B, Liu P, Li T, Si W, Xiu J, Liu H (2014). Package of NDV-pseudotyped HIV-Luc virus and its application in the neutralization assay for NDV infection. PLoS One.

